# Alternative glycosylation controls endoplasmic reticulum dynamics and tubular extension in mammalian cells

**DOI:** 10.1126/sciadv.abe8349

**Published:** 2021-05-07

**Authors:** Despoina Kerselidou, Bushra Saeed Dohai, David R. Nelson, Sarah Daakour, Nicolas De Cock, Zahra Al Oula Hassoun, Dae-Kyum Kim, Julien Olivet, Diana C. El Assal, Ashish Jaiswal, Amnah Alzahmi, Deeya Saha, Charlotte Pain, Filip Matthijssens, Pierre Lemaitre, Michael Herfs, Julien Chapuis, Bart Ghesquiere, Didier Vertommen, Verena Kriechbaumer, Kèvin Knoops, Carmen Lopez-Iglesias, Marc van Zandvoort, Jean-Charles Lambert, Julien Hanson, Christophe Desmet, Marc Thiry, Kyle J. Lauersen, Marc Vidal, Pieter Van Vlierberghe, Franck Dequiedt, Kourosh Salehi-Ashtiani, Jean-Claude Twizere

**Affiliations:** 1 Laboratory of Viral Interactomes, GIGA Institute, University of Liege, Liege, Belgium.; 2 Laboratory of Gene expression and Cancer, GIGA Institute, University of Liege, Liege, Belgium.; 3 Division of Science and Math, New York University Abu Dhabi, Abu Dhabi, UAE.; 4 Center for Genomics and Systems Biology (CGSB), New York University Abu Dhabi, Abu Dhabi, UAE.; 5 TERRA Teaching and Research Centre, University of Liege, Liege, Belgium.; 6 The Donnelly Centre, University of Toronto, Toronto, ON, Canada.; 7 Department of Molecular Genetics, University of Toronto, Toronto, ON, Canada.; 8 Lunenfeld Tanenbaum Research Institute, Mount Sinai Hospital, Toronto, ON, Canada.; 9 Plant Cell Biology, Biological and Medical Sciences, Oxford Brookes University, Oxford, UK.; 10 Department of Biomolecular Medicine and Center for Medical Genetics, Ghent University, Ghent, Belgium.; 11 Cancer Research Institute Ghent (CRIG), Ghent, Belgium.; 12 GIGA-I3 Unit, GIGA Institute, University of Liege, Liege, Belgium.; 13 GIGA-Cancer Unit, GIGA Institute, University of Liege, Liege, Belgium.; 14 Laboratory of Excellence Distalz, INSERM Unit 1167, Pasteur Institute of Lille, Lille, France.; 15 Metabolomics Expertise Center, Center for Cancer Biology, VIB Center for Cancer Biology, Leuven, Belgium.; 16 de Duve Institute, Catholic University of Louvain, Brussels, Belgium.; 17 Microscopy CORE Lab, Maastricht Multimodal Molecular Imaging Institute, Maastricht University, Maastricht, Netherlands.; 18 Department of Cell Biology, School for Cardiovascular Diseases (CARIM), School for Nutrition and Translational Research in Metabolism (NUTRIM), School for Mental health and Neuroscience (MHeNS), and School for Oncology and Developmental Biology (GROW), Maastricht University, Maastricht, Netherlands.; 19 GIGA-Molecular Pharmacology, University of Liege, Liege, Belgium.; 20 Laboratory of cell and tissue Biology, GIGA-Neurosciences, University of Liege, Liege, Belgium.; 21 Biological and Environmental Sciences and Engineering Division (BESE), King Abdullah University of Science and Technology (KAUST), Thuwal, Kingdom of Saudi Arabia.; 22 Center for Cancer Systems Biology (CCSB), Dana-Farber Cancer Institute, Boston, MA, USA.; 23 Department of Genetics, Blavatnik Institute, Harvard Medical School, Boston, MA, USA.

## Abstract

The endoplasmic reticulum (ER) is a central eukaryotic organelle with a tubular network made of hairpin proteins linked by hydrolysis of guanosine triphosphate nucleotides. Among posttranslational modifications initiated at the ER level, glycosylation is the most common reaction. However, our understanding of the impact of glycosylation on the ER structure remains unclear. Here, we show that exostosin-1 (EXT1) glycosyltransferase, an enzyme involved in *N*-glycosylation, is a key regulator of ER morphology and dynamics. We have integrated multiomics and superresolution imaging to characterize the broad effect of *EXT1* inactivation, including the ER shape-dynamics-function relationships in mammalian cells. We have observed that inactivating *EXT1* induces cell enlargement and enhances metabolic switches such as protein secretion. In particular, suppressing *EXT1* in mouse thymocytes causes developmental dysfunctions associated with the ER network extension. Last, our data illuminate the physical and functional aspects of the ER proteome-glycome-lipidome structure axis, with implications in biotechnology and medicine.

## Teaser

Integration of multiomics and imaging identifies a glycosyltransferase enzyme regulating endoplasmic reticulum sizes.

## INTRODUCTION

The endoplasmic reticulum (ER) is one of the largest organelles of eukaryotic cells (*1*). It facilitates communication with other intracellular organelles through its connection to the nuclear envelope and regulates interactions with the external environment via the secretion of proteins, polysaccharides, and lipids (*2*).

The ER is involved in numerous cellular processes, from lipid turnover to protein secretion and glycosylation. Part of the metabolic flexibility of the ER is mediated by its dynamic and adaptable shape (*3*). During normal cell homeostasis, the ER is a complex network of tubules and flat matrices that are in continuous motion. These substructures form a three-dimensional, regularly shaped network, which derives its form from the lipid bilayer and different groups of membrane-associated proteins (*4*). High-curvature regions, such as ER tubules and edges of ER sheets, are built by the oligomerization of hydrophobic hairpin domain–containing reticulons (RTNs) and receptor expression–enhancing proteins (REEPs) (*5*, *6*). Flat matrices are formed by atlastin (ATL) guanosine triphosphatase (GTPase)–ER membrane associations; these proteins dimerize in opposing layers to hold lipid bilayers in this conformation (*7*). The curvature of flat matrices is mediated by the luminal bridging cytoskeleton-linking membrane protein 63 (*8*).

It is currently believed that cooperation between ER shape and luminal dynamics dictates ER functions (*9*). ER sheets are the primary sites for translation, translocation, and folding of integral membrane-bound and secreted proteins, while ER tubules are thought to be involved in other ER functions such as lipid synthesis and interactions with other organelles (*5*, *10*). Cells actively adapt their ER tubule/sheet balance and dynamics to coordinate its morphology and function, in accordance with cellular demands (*11*). However, the molecular mechanisms underlying overall maintenance and flexibility of the ER network remain poorly characterized.

One of the key roles the ER plays for the cell is the secretion of extracellular products and glycosylation of proteins. Our understanding of the mechanisms of glycosylation is now advanced enough to engineer cell lines to confer customized glyco motifs for biotechnological and medicinal applications. However, our understanding of how these motifs affect the intracellular dynamics of cellular homeostasis is limited. Glycosylation proceeds by the synthesis of glycans and attachment to the acceptor peptide, which is initiated in the ER and terminates in the Golgi apparatus (*12*). Glycosylation is well known to regulate the physical properties of different glycolipid and glycoprotein biopolymers at the surface of mammalian cells by controlling plasma membrane and cell coat morphologies (*13*). The impact of intracellular ER membrane protein component glycosylation, their interactions with membranes, and their contributions to cellular dynamics is entirely unknown. While permanent interactions between membrane curvature proteins are sufficient to form the basic ER structure (*9*, *14*), protein-protein interactions and posttranslational modifications may participate in its dynamic shaping. It is, therefore, essential to decipher how glycosylation affects ER dynamics within the cell in addition to defining the sequential steps leading to final glycosylation species of extracellular proteins.

Synthesis of glycans and their attachment to proteins occurs by the sequential activities of glycosyltransferases and glycosidases that compete for activated glycans and overlapping substrates (*12*). Protein *N*-glycosylation occurs in the ER lumen and is catalyzed by the oligosaccharyltransferase (OST) complex, which is composed of eight proteins in metazoans [ribophorin, defender against cell death 1 (DAD1), tumor suppressor candidate 3 (Tusc3), OST4, transmembrane protein 258 (TMEM258), OST48, and catalytic subunits STT3 oligosaccharyltransferase complex catalytic subunit A (STT3A) and STT3B] (*15*). The final composition of oligosaccharide chains bound to glycoproteins depends not only on localization and abundance of these enzymes but also on the availability and heterogeneity of sugar substrates. Exostosin-1 (EXT1) is an ER-resident glycosyltransferase involved in the polymerization of heparan sulfate (HS) (*16*). HS molecules are found in all animal tissues and play a key role in many biological activities, including development and cancer. While investigating the role of EXT1 in thymocyte development and cancer, we found that reduction of the EXT1 protein results in global changes in cellular homeostasis, including cell size, organelle shapes and interactions, and cellular metabolism. We show that reprogramming of glycan moieties by reduction of this regulator can profoundly change ER structure concurrent with global metabolic shifts in protein and membrane lipid synthesis in cells.

## RESULTS

### Developmental defects following *EXT1* inactivation are mediated by its genetic interactions

**Fig. 1:**
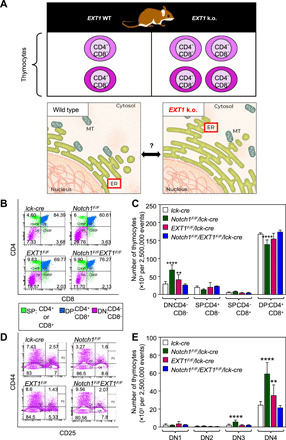
Developmental defects following *EXT1* inactivation are mediated by its genetic interactions. (**A**) Framework to study the role of ER-resident EXT1 in thymocyte development. MT, mitochondria. (**B**) Representative fluorescence-activated cell sorting (FACS) plots showing the surface phenotype of CD4 and CD8 T cells in thymocytes. Cell percentages are shown in quadrants. (**C**) The absolute number of thymocytes (of 2,500,000 total events) showing the surface receptor expression of DN, single positive (SP) and double positive (DP) populations. *n* = 6 mice (*lck-cre*, *Notch1^F/F^/lck-cre*, *EXT1^F/F^/lck-cre*, and *Notch1^F/F^/EXT1^F/F^/lck-cre*). One-way ANOVA: **P < 0.01, ****P < 0.0001. (**D**) Representative FACS plots showing the surface expression of CD44 and CD25 markers in DN populations. Cell percentages are shown in quadrants. (**E**) The absolute number of DN1, DN2, DN3, and DN4 cells (of 2,500,000 total events). One-way ANOVA: **P < 0.01, ****P < 0.0001. See also fig. S1.

In a systematic interactome study, we previously showed that EXT1, an ER-resident type II transmembrane glycosyltransferase, interacts with Notch1, a type I transmembrane receptor that is frequently mutated in cancers (*17*). Notch1 is essential for the development of numerous cell types, including thymocytes (*18*, *19*). We thus hypothesized that EXT1 might play a physiological role in T cell development, potentially associated with its glycosyltransferase function and ER residency (Fig. 1A). Because homozygous *EXT1*-null mice die at embryonic day 8.5 (*20*), we crossed *EXT1^F/F^* (*21*) mice with mice expressing the Cre recombinase under the control of *lck* proximal promoter (*22*) to specifically target *EXT1* in early developing thymocytes. We found that *EXT1* inactivation affects the early stage of thymocyte development, with a significant accumulation of immature double-negative CD4^−^, CD8^−^ cells (DN) (*P* < 0.01; Fig. 1, B and C). We also used a *Notch^F/F^* line (*23*) to generate a conditional knockout (k.o.) of *Notch1* or both *EXT1 and Notch1* genes simultaneously (fig. S1, A and B). As previously shown (*19*), *Notch1* inactivation leads to the accumulation of DN, at a higher extend compared to *EXT1* k.o. (*P* < 0.0001; Fig. 1, B and C). In both cases, *Notch1* or *EXT1* inactivation affects the late stages of thymocyte developmental stages DN3 and DN4 (Fig. 1, D and E). Unexpectedly, transgenic mice with thymic inactivation of both genes exhibit a normal phenotype, suggesting a genetic suppression interaction between *Notch1* and *EXT1* in thymocytes (Fig. 1, B to E). Because the altered phenotype in *Notch1*-deficient thymocytes was rescued by *EXT1* k.o., we concluded that *EXT1* may act as a functional suppressor partner of the *Notch1* receptor in vivo.

### Cancer dependency to *EXT1* expression is associated with perturbations of ER structures

**Fig. 2:**
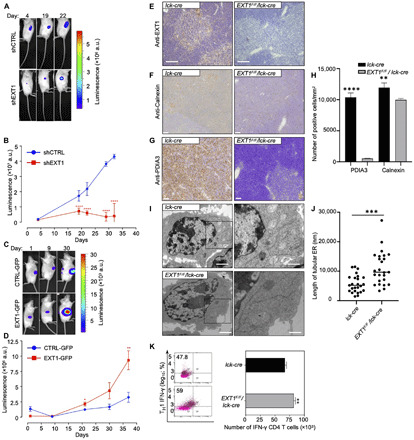
Cancer dependency to EXT1 expression is associated with perturbations of ER structures. (**A** and **B**) Follow-up of the tumor progression via bioluminescence after injection of 2 × 10^6^ control and shEXT1 Jurkat cells at opposites sites of NOD/SCID mice. a.u., arbitrary units. ****P < 0.0001. (**C** and **D**) As in (A) and (B) for CTRL–GFP (green fluorescent protein) and EXT1-GFP cells. One-way analysis of variance (ANOVA), *P < 0.05, **P < 0.01, and ****P < 0.0001 (*n* = 6 to 10 mice per group). (**E**) Immunohistochemistry staining of EXT1 protein in thymus of *lck-cre* (left) and *EXT1*^F/F^/*lck-cre* (right) mice. Scale bars, 2 μm. (**F**) As in (E) but immunohistochemistry staining with anti-Calnexin antibody. Scale bars, 2 μm. (**G**) As in (E) but immunohistochemistry staining with anti–protein disulfide isomerase family A member 3 (PDIA3) antibody. Scale bars, 2 μm. (**H**) Quantifications of NanoZoomer Digital Pathology Image (ndpi) slide scans using QuPath 0.2.0-m8. Positive cells per square millimeter from indicated conditions are plotted. One-way ANOVA, **P < 0.01 and ****P < 0.0001 (*n* = 4 scan area per staining, five mice per group). (**I**) TEM of ER in activated T cells from murine peripheral lymph nodes and spleen. Scale bars, 2 μm. Boxed region illustrates the ER. (**J**) Quantification of the length of tubular ER. One-way ANOVA, ***P < 0.001 (*n* = 25 cells, 6 to 10 mice per group). (**K**) The percentages of interferon-γ (IFN-γ) in T helper 1 cell (T_H_1) from *lck-cre* and *EXT1*^F/F^/*lck-cre* mice are shown in quadrants. Percentages of IFN-γ^+^ cells are shown in quadrants. Bar graphs represent mean number ± SD. One-way ANOVA, **P < 0.01 (*n* = 6 to 10 mice per group). See also figs. S1 and S2.

The unexpected phenotype generated by *Notch1* and *EXT1* double k.o. allowed us to hypothesize that *EXT1* could be a candidate synthetic lethal (SL) (*24*) or synthetic dosage lethal (SDL) (*25*) gene with activated oncogenic Notch1. To test the SDL hypothesis, we knocked down or overexpressed *EXT1* in Jurkat (fig. S1, C and D), a T cell acute lymphoblastic leukemia (ALL) cell line, which has altered Notch1 signaling (fig. S1, E and F). Dosage variations did not influence Jurkat cell proliferation (fig. S1G). However, when injected into nonobese diabetic/severe combined immunodeficient (NOD/SCID) mice (*26*), we observed a notable and significant reduction in tumorigenicity following *EXT1* knockdown (k.d.) (*P* < 0.0001; Fig. 2, A and B). Concurrently, overexpression of EXT1 was found to cause more tumor burden than control Jurkat T-ALL cells (Fig. 2, C and D), demonstrating a dosage lethality effect of EXT1 in the Jurkat T cell model. To test the SL hypothesis, we interrogated the gold standard SL gene pairs across different cancer types (*24*, *25*). In these cancer patient cell lines, we did not observe any SL interaction between *EXT1* and *Notch1*, or *EXT1* and the Notch1 ubiquitin ligase encoding gene *FBXW7*. However, *EXT1* and *Notch1* do synthetically interact with several shared genes, including important oncogenes: *KRAS*, *PTEN*, *BRCAC2*, and *MYC* (fig. S1H). *EXT1* also appears as a clinically significant hub, for which down-regulation by short hairpin RNA (shRNA) presented numerous SL interactions relevant for various cancer types (fig. S1H). An exploration of The Cancer Genome Atlas (TCGA) for somatic mutations in different cancer cell lines and tumors also highlighted *EXT1* as a clinically relevant hub (fig. S1, I and J). These findings suggest that *EXT1* is a genetic suppressor of *Notch1* and a potential precision therapeutic target in cancers for which Notch1 and other selected oncogenes are activated.

We next sought to investigate the molecular mechanisms underlying the identification of *EXT1* as a suppressor hub. Because the EXT1 protein localizes predominantly in the ER (*27*), we first assessed ER phenotypes of the thymus from mutant mice with inactivation of *EXT1* (*EXT1^F/F^/lck-cre*) (Fig. 2E). We used immunohistochemistry to examine the expression of the ER-resident molecular chaperones Calnexin and protein disulfide isomerase family A member 3 (PDIA3) and observed a notable reduction in the expression of both markers in structural cells of the thymus from conditional *EXT1 k.o.* compared to control mice (Fig. 2, F to H). To gain information about the consequences of *EXT1 k.o.* in the thymus, we analyzed mature lymphocytes migrating from the thymus. Transmission electron microscopy (TEM) analysis highlighted an unusual elongated ER morphology in activated CD4^+^ T cells from *EXT1 k.o.* mice (Fig. 2, I and J), with a concomitant increase in the T helper 1 interferon-γ (IFN-γ)–producing cell population (Fig. 2K). The results suggest an important role of EXT1 in ER organization in T cells.

### *EXT1* down-regulation causes ER extension and cell size increase

**Fig. 3:**
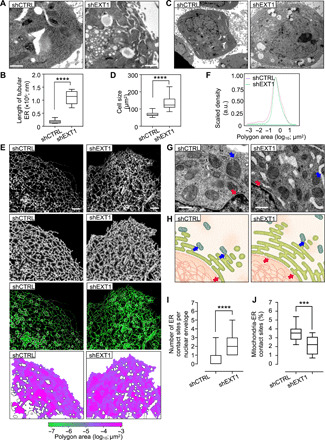
EXT1 down-regulation causes ER extension and cell size increase. (**A**) TEM of the ultrastructure ER of HeLa cells. Scale bars, 2 μm. (**B**) Quantification of the length of tubular ER (in nanometers) per cell (*n* = 10 cells, three independent experiments). Box plot indicates that the mean and whiskers show the minimum and maximum values. One-way ANOVA, ****P < 0.0001. (**C**) TEM of HeLa shCTRL and shEXT1 cells. Scale bars, 2 μm. (**D**) Quantification of the cell area (*n* = 19 to 22 cells, three independent experiments; table S1). One-way ANOVA, ****P < 0.0001. (**E**) Confocal fluorescence of Cos7 cells shCTRL or shEXT1 transiently expressing mEmerald-Sec61b. From top to bottom: original image, skeleton, overlay of the skeleton (purple), the cisternae (white) and the original image (green), polygonal regions map, and color-coded by size. (**F**) Quantitative analysis based on the skeletonization model of Cos7 cells expressing mEmerald-Sec61b under shCTRL and shEXT1 condition. Polygon area (log_10_) in *x* axis is plotted against scaled density in *y* axis (*n* = 19 to 24). (**G**) TEM of ER-mitochondria and ER–nuclear envelope contact sites in HeLa shCTRL and shEXT1 cells. Arrows in blue and red highlight the contact sites. The nuclear envelope–ER contact sites increase and the number of mitochondria-ER contact sites decreases following EXT1 depletion. Scale bars, 500 nm. (**H**) Schematic representation of the ER–other organelle contact sites, as used for the statistical analysis of the different parameters. (**I**) Quantification of the ER–nuclear envelope contact sites in box plot indicating that the mean and whiskers show the minimum and maximum values (*n* = 10 to 18 cells, three independent experiments). One-way ANOVA, ****P < 0.0001. (**J**) As in (I) but percentage of ER-mitochondria contact sites (*n* = 10). One-way ANOVA, ***P < 0.001. See also figs. S2 and S3.

The elongated morphology of ER in *EXT1 k.o.* mice thymocytes was unexpected. To rule out cell line–specific effects, we assessed the ultrastructural ER morphology in HeLa, human embryonic kidney (HEK) 293, and Jurkat cells. Following *EXT1* k.d., we observed a dramatic elongation of ER tubules in all cell lines; for HeLa cell line, an average length of 109.6 ± 25.3 μm is compared to 19.0 ± 8.0 μm in control cells (Fig. 3, A and B, and fig. S2, A to E). The depletion of other members of the exostosin family (*EXT2* and *EXTL1-3*) did not lead to similar ER changes (fig. S2, F to H). Probably as a consequence of ER extension, the cell area increased by ~2-fold in *EXT1 k.d.* cells compared to controls (133.9 ± 36.8 and 68.5 ± 12.5 μm^2^, respectively) (Fig. 3, C and D). Cell size is of fundamental importance to all biological processes, and it is strictly regulated to keep a balance between growth and division. We did not observe any significant effect on proliferation following *EXT1 k.d.* (fig. S3, A and B), suggesting that *EXT1 k.d.* cells might have undergone an important adaptive change of the size threshold following ER extension and internal cellular architecture rearrangement (Fig. 3, A and C).

To analyze the ER luminal structural rearrangements, we quantified ER membrane structures marked with SEC61 translocon subunit beta (SEC61b) by confocal microscopy and a segmentation algorithm that excludes insufficient fluorescent intensity. This strategy generates a single-pixel-wide network to allow the quantification of individual tubule morphological features. The tubular ER network was altered in *EXT1 k.d*. cells and exhibited a denser and more reticulated phenotype in comparison to controls (Fig. 3E). Measurements of the polygonal area of the ER tubular network in these cells were 0.778 μm^2^, which is a reduction from 0.946 μm^2^ in controls (Fig. 3F). Other tubular and cisternal ER metrics were unaffected (fig. S3, C to E), suggesting that the dense tubular network might relate to a more crowded ER lumen.

We next analyzed ER interactions with other organelles and counted significantly more peripheral ER–nuclear envelope (2.3 ± 1.2 versus 0.6 ± 0.9) and less ER-mitochondria (21.6 ± 10.2 versus 35.4 ± 9.3) interactions in HeLa *EXT1 k.d*. compared to control cells (Fig. 3, G to J). The latter was unexpected given the ~5.7-fold increase in ER length (Fig. 3B). However, it was found to correlate with an impaired calcium flux in those cells (fig. S3, F and G), suggesting that cells undergo a metabolic switch following *EXT1 k.d*.

### EXT1 reduction induces Golgi reorganization and a metabolic switch

**Fig. 4:**
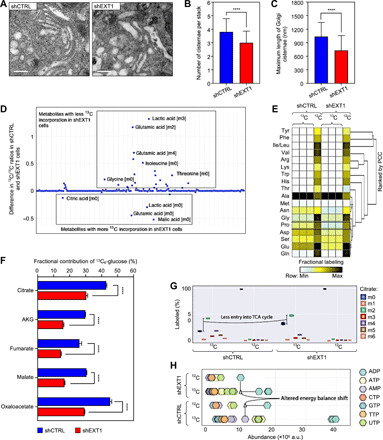
EXT1 reduction induces Golgi reorganization and a metabolic switch. (**A**) TEM of Golgi apparatus in HeLa shCTRL and shEXT1 cells. Silencing *EXT1* results in reduced number and increased size of Golgi cisternae/stacks. Scale bars, 500 nm. (**B** and **C**) The number of Golgi cisternae/stacks (B) and maximum length of individual Golgi cisternae (in nanometers) (C) were quantified on the basis of TEM images (*n* = 20; table S1). One-way ANOVA, ****P < 0.0001. (**D**) Scatterplot of raw cell abundance profile of metabolites. (**E**) Heatmap *z* score represents fractional labeling of metabolites. Metabolites were clustered using one-minus Spearman’s rank correlation. PCC, Pearson’s Correlation Coefficient. (**F**) Fractional contribution from ^13^C_6_-glucose to TCA metabolites (*n* = 3). One-way ANOVA, ****P < 0.0001. AKG, α-ketoglutarate. (**G**) Isotopomer distribution of citrate derivatives into the TCA cycle in HEK293 cells (*n* = 3). (**H**) Cell abundance of nucleotides showing altered energy balance shift. AMP, adenosine 5′-monophosphate; TTP, thymidine 5′-triphosphate. See also fig. S4.

Although EXT1 is an ER-resident protein, it is also found in the Golgi apparatus, where it forms a catalytic heterodimer enzyme that polymerizes the elongation of HS chains by sequential addition of glucuronic acid and *N*-acetylglucosamine (GlcNAc) (*28*). In addition to the notable changes in ER structure, TEM ultrastructural examination of *EXT1 k.d*. cells revealed structural changes in the Golgi apparatus size and shape (Fig. 4A). The number of Golgi cisternae per stack was reduced (3.0 ± 0.9 in *EXT1 k.d.* compared to 3.8 ± 1.0 in control cells; Fig. 4B), and stacks were dilated and shorter in length (729.2 ± 329.0 in *EXT1 k.d.* compared to 1036.0 ± 312.0 nm in control cells; Fig. 4C). Modified Golgi morphology combined with the reduction in ER-mitochondria interactions points toward global metabolic changes in *EXT1 k.d.* cells.

To assess the implications of EXT1 in cellular metabolism, we used two different strategies. First, we generated transcriptomic data (*17*) from cells treated with *EXT1* small interfering RNA (siRNA) and control cells to reconstruct two in silico flux balance analysis (FBA) models using constraint-based reconstruction analysis (COBRA) (*29*) tools and the human *RECON2* metabolic model (*29*). We found, respectively, 34 and 39 reactions uniquely active in the *EXT1 k.d*. or control models when the production of biomass was optimized (fig. S4A). These reactions are involved in the tricarboxylic acid (TCA) cycle, glycerophospholipid metabolism, pyruvate, methane, and sphingolipid metabolism (fig. S4B). Second, we performed a high-throughput metabolomic analysis of the relative abundance and fractional contribution of intracellular metabolites from major metabolic pathways in living *EXT1 k.d.* compared to control cells. We did not observe significant changes in glycolysis between *EXT1 k.d*. and control cells. However, in agreement with our in silico FBA, we found that several nucleotides, amino acids, and metabolites from the TCA cycle were dysregulated in *EXT1 k.d*. cells (Fig. 4, D to H, and fig. S4, C to E).

The fractional contribution of glucose carbons into these pools of metabolites was also decreased in *EXT1 k.d*. cells (Fig. 4, E and F). For instance, the fractional contributions of citric acid (change, 12.51%; *P* < 0.001), α-ketoglutarate (change, 13.87%; *P* < 0.0001), fumarate (change, 11.61%; *P* < 0.001), malate (change, 13.74%; *P* < 0.0001), and oxaloacetate (change, 15.97%; *P* < 0.0001) were significantly reduced in *EXT1 k.d*. cells (Fig. 4F). Isotopologue profile analysis of TCA intermediates suggested that mitochondria were in a less oxidative mode of action in *EXT1 k.d*. cells, as m03, m04, m05, and m06 of citric acid were much lower in abundance (Fig. 4G and fig. S4E). In contrast, metabolite pools of the pentose phosphate pathway, the m05 of different nucleotides [adenosine triphosphate (ATP), uridine triphosphate (UTP), guanosine triphosphate (GTP), and cytidine triphosphate (CTP)], and the energy charge were increased in the *EXT1 k.d*. cells (Fig. 4H and fig. S4, F and G). Together, these findings indicate a higher de novo synthesis and consumption rate of nucleotides necessary for the synthesis of sugar intermediates used in protein glycosylation [i.e., uridine diphosphate (UDP)–GlcNAc] in the *EXT1 k.d*. cells.

### *EXT1 k.d*. causes changes in the molecular composition of ER membranes

**Fig. 5:**
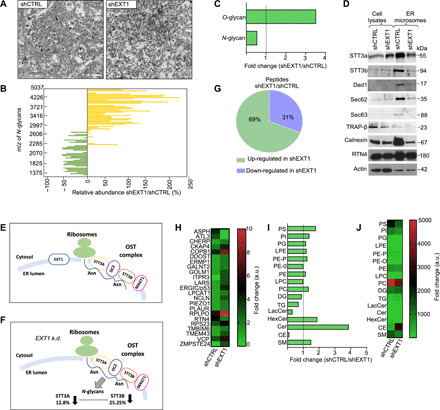
*EXT1 k.d*. causes changes in the molecular composition of ER membranes. (**A**) TEM of ER microsomes isolated from HeLa cells. Scale bars, 1 μm. (**B**) Glycomic analysis of microsomes. Relative abundance of each *N*-glycan in shEXT1 versus shCTRL microsomes. The variations are plotted by *N*-glycan mass. (**C**) As in (B), the bars indicate the fold change of the total *O*- and *N*-glycan intensities. (**D**) Expression of OST complex subunits (STT3a, STT3b, and Dad1), other translocon members (Sec62, Sec63, and Trap-β), and ER constitutive markers Calnexin and RTN4 in microsomes. (**E** and **F**) Schematic representation of the OST complex for which catalytic subunits STT3A and STT3B are less glycosylated following *EXT1 k.d.* MAGT1, magnesium transporter 1. (**G**) Quantitative proteomic analysis of microsomes. Pie chart illustrates the number of up-regulated and down-regulated proteins. (**H**) Heatmap to quantify 23 ER integral proteins. ASPH, aspartate beta-hydroxylase; CHERP, calcium homeostasis endoplasmic reticulum protein; CKAP4, cytoskeleton associated protein 4; COPB1, COPI coat complex subunit beta 1; DDOST, dolichyl-diphosphooligosaccharide–protein glycosyltransferase non-catalytic subunit; ERMP1, endoplasmic reticulum metallopeptidase 1; GALNT2, polypeptide N-acetylgalactosaminyltransferase 2; GOLM1, Golgi membrane protein 1; ITPR3, inositol 1,4,5-trisphosphate receptor type 3; LARS, leucyl-TRNA synthetase 1; ERGIC/P53, endoplasmic reticulum-Golgi intermediate compartment 53 KDa protein; LPCAT1, lysophosphatidylcholine acyltransferase 1; NCLN, nicalin; PIEZO1, piezo type mechanosensitive ion channel component 1; PLAUR, plasminogen activator, urokinase receptor; RPLPO, 60S acidic ribosomal protein PO; RPS23, ribosomal protein S23; TMBIM6, transmembrane BAX inhibitor motif containing 6; TMEM43, transmembrane protein 43; VCP, valosin containing protein; ZMPSTE24, zinc metallopeptidase STE24. (**I**) Lipidomic analysis of different lipid species as found in microsomes. Bars indicate the fold change of the total intensity (a.u.). (**J**) As in (I), alternatively graphed in a heatmap. See also fig. S5.

To understand the molecular mechanisms of the EXT1-mediated cellular metabolic changes observed above, we isolated the ER microsomes from *EXT1 k.d*. and control cells. TEM revealed that, in the absence of EXT1, the structure of ER membranes was modified, as vesicle-like fragments were observed compared to the normal heterogeneous microsomes in control cells (Fig. 5A).

On the basis of this observation, we comprehensively compared the glycome, proteome, and lipidome profiles of those ER membranes in control and *EXT1 k.d*. cells. Glycome analysis using matrix-assisted laser desorption/ionization–time-of-flight mass spectrometry (MALDI-TOF MS) enabled absolute and relative quantification of glycoprotein *N*- and *O*-glycan abundances, respectively, in these cell lines. The k.d. of *EXT1* did not induce the appearance of new glycan species on membrane proteins (fig. S5A). However, we observed a significant shift toward higher–molecular weight *N*-glycans and more *O*-glycosylation compared to control ER membranes (Fig. 5, B and C). The total relative amount of *N*-glycans was reduced, consistent with the lower cellular abundance of UDP-GlcNAc (Fig. 5C and fig. S5B). This deregulation appears to occur, at least in part, at the level of the first step during protein *N-*glycosylation, which involves the OST complex. Microsomes isolated from *EXT1 k.d*. cells had reduced amounts of OST complex proteins STT3A, STT3B, and Dad1 (Fig. 5D).

*N*-glycosylation in eukaryotes is cotranslational (*30*). In agreement with the finding that *EXT1 k.d*. impairs *N*-glycosylation, we observed that some members of the translocon complex (Sec62 and Sec63), the translocon-associated protein complex (TRAP), and the *N*-glycosylation quality control protein Calnexin were found to be reduced in *EXT1 k.d*. cells compared to controls (Fig. 5D). Using a tandem MS (MS/MS) analysis followed by glycopeptide identification, we found that *EXT1 k.d*. specifically reduced the *N*-glycosylation of asparagine (N) residues N548 and N627 in STT3A and STT3B, respectively, which are the catalytic subunits of the OST complex (Fig. 5, E and F). Although the role of *N*-glycosylation of human STT3A and STT3B is still unknown, in yeast, *N*-glycosylation of the ortholog Stt3 mediates the assembly of the OST subcomplexes via interaction with wheat germ agglutinin-binding protein (Wbp1) and suppressor of a Wbp1 mutation (Swp1) (*31*).

MS/MS proteomic analysis also identified 226 proteins that were differentially abundant in ER membranes of *EXT1 k.d*. cells, including 23 ER-resident proteins. Specifically, RTN4 and ATL3 ER-shaping proteins were found in lower abundance in *EXT1 k.d*. cells (Fig. 5, G and H). However, valosin-containing protein (VCP), an adenosine triphosphatase involved in lipid recruitment during ER formation, and a glycan-binding component of the ER-Golgi intermediate compartment that is involved in ER reorganization, ERGIC/p53, were in higher abundance (Fig. 5H). Consistent with the increase in *O*-glycans (Fig. 5B), ER membranes from *EXT1 k.d*. cells were also found to have higher amounts of *N*-acetylgalactosamine transferase 2 (GALNT2) (Fig. 5H) and an overall higher glycosyltransferase activity in ER microsomes (fig. S5C). These results confirm that the ER proteome, including shaping proteins and ER enzymes, is deregulated following *EXT1 k.d*.

Examination of lipid classes in ER microsomes also highlighted significant changes following EXT1 depletion (Fig. 5, I and J). The most significant increase was observed in cholesterol esters (CEs), which were ~9-fold higher in *EXT1 k.d*. membranes compared to control (Fig. 5, I and J). Changes were also observed in phospholipids such as phosphatidylcholine (PC), phosphatidylserine (PS), and sphingomyelin (SM) (Fig. 5, I and J). We concluded that, in the absence of EXT1, ER membrane lipid composition is modified toward structural fluidity.

### EXT1 localizes in ER tubules and sheet matrices

**Fig. 6:**
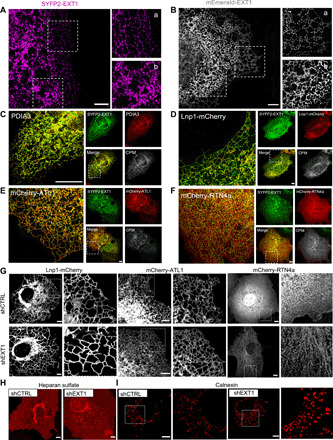
EXT1 localizes in ER tubules and sheet matrices. (**A** and **B**) STED (A) and SIM (B) images of Cos7 cells expressing SYFP2-EXT1 and mEmerald-EXT1, respectively. Boxed regions illustrate the tubular (subpanel a) and the cisternal (subpanel b) ER. Scale bars, 4 μm. (**C**) Confocal fluorescence microscopy of Cos7 cells transiently expressing SYFP2-EXT1 (green) and endogenous ER marker PDIA3 (red). The subpanels show the individual and merged channels and the colocalized pixel map (CPM). Scale bar, 4 μm. (**D** to **F**) As in (C) but coexpression of SYFP2-EXT1 (green) and indicated ER markers (red). Scale bars, 4 μm. (**G**) Live imaging of shCTRL and shEXT1 Cos7 cells stably expressing indicated ER markers. Scale bars, 4 μm. Boxed region illustrates the ER. (**H**) HS endogenous staining (red) of Cos7 cells. Scale bars, 5 μm. (**I**) Endogenous staining (red) of Cos7 cells with Calnexin antibody. Boxed regions magnified illustrate a zoom of circular, vesicle-like structures that appear following EXT1 k.d. Scale bars, 4 μm. See also fig. S6.

The above results suggest a role of EXT1 in the maintenance of the ER structure. Previous studies have shown that EXT1 localizes predominantly to the ER (*16*). However, whether EXT1 localizes in the ER tubules or sheet matrices was not investigated because of the spatial limitations of optical microscopy. To precisely characterize EXT1 localization in ER structures, we performed superresolution (SR) imaging with EXT1 tagged with strongly enhanced yellow fluorescent protein 2 (SYFP2) and mEmerald, two fluorophores with different photostability properties. Using two SR technologies, stimulated emission depletion (STED) and structured illumination microscopy (SIM), we found that EXT1 localized in dense sheets and peripheral ER tubules (Fig. 6, A and B, and fig. S6A). EXT1 largely colocalized with the ER luminal marker PDIA3 (Fig. 6C) and, to lesser extent, with lectin chaperone Calnexin and Golgi marker GM130 (fig. S6, B to D). EXT1 perfectly colocalized with ER-shaping proteins Lunapark1 (Lnp1), ATL1, and RTN4a in tubules and the ER three-way junctions (Fig. 6, D to F, and fig. S6D).

To assess whether *EXT1 k.d*. might affect ER luminal dynamics, we analyzed the dynamic motion of ER tubules and three-way junctions by tracking the trajectories of ATL1 and Lnp1 proteins using live imaging (movies S1 to S4). In validated Cos7 *EXT1 k.d*. cells (fig. S6E), the ER periphery morphology is asymmetrically dispersed compared to controls cells. While Lnp1 and ATL1 markers showed significant increase in the tubular ER polygon area (fig. S6F), RTN4a showed increased membranous localization following *EXT1* k.d. (Fig. 6G). We quantified the ratio between the ER tubules and three-way junctions, which indicated that the ER fusion rate was not affected following k.d. of *EXT1* (fig. S6G). Next, we adapted a previously described single-molecule localization algorithm (*32*) to reconstruct the diffusivity and velocities at the three-way junctions (fig. S6, H to J). We computed particle distributions, trajectories, and velocities and found ATL1 to have a higher diffusivity and instantaneous velocity than Lnp1 (fig. S6, H to J), consistent with their respective localizations in the ER tubules and three-way junctions. The maximum tubular motion observed here (velocity of ~3 μm/s) was lower than the luminal motion in previous observations (10 to 40 μm/s) (fig. S6K) (*32*). *EXT1* reduction by k.d. did not affect tubule motion, suggesting that the ER morphology changes in *EXT1 k.d*. cells might result from luminal flow changes, potentially driven by intracellular redistribution of HS (Fig. 6H).

The molecular chaperone Calnexin, which assists protein folding in the ER, exhibited an aggregation pattern in *EXT1 k.d*. cells (Fig. 6I and fig. S6N), which might result in decreased movement of molecules through the ER lumen. To assess how a reduced polygonal area following *EXT1 k.d*. might influence ER luminal protein mobility and network continuity, we quantified the relative diffusion and active transport through the lumen of an ER lumen marker PA–GFP–KDEL (photoactivable–green fluorescent protein–lysine-aspartic acid-glutamic acid-leucine). Its signal was spread throughout the entire ER network, demonstrating that the continuity of ER was not affected in *EXT1 k.d*. cells (movies S5 and S6). However, we observed a significantly higher dynamic of fluorescence intensity in regions close to the nucleus (fig. S6, L and M, at 8, 12, and 16 μm), suggesting that the structural rearrangements of the ER following *EXT1 k.d*. actively participate in luminal protein transport. Together, these data demonstrate that *EXT1 k.d*. induces ER morphological changes that impair protein movement through the ER.

### *EXT1 k.d*. results in increased secretory cargo trafficking

**Fig. 7:**
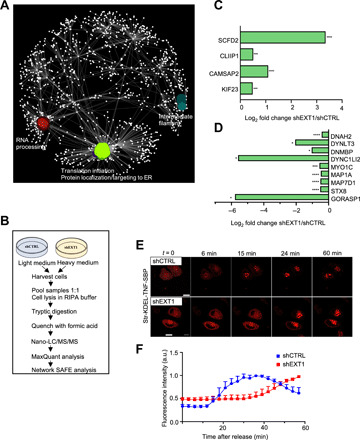
*EXT1 k.d*. results in increased secretory cargo trafficking. (**A**) SAFE analysis of EXT1 interactome in ER microsomes. (**B**) Schematic representation of the SILAC workflow. (**C** and **D**) Up-regulated (C) and down-regulated (D) proteins involved in anterograde and retrograde transport. (C) CAMSAP2, calmodulin regulated spectrin associated protein family member 2; CLIP1, CAP-gly domain-containing linker protein 1; KIF23, kinesin family member 23; SCFD2, Sec1 family domain containing 2. (D) DNAH2, dynein axonemal heavy chain 2; DNMBP, dynamin binding protein; DYNC1LI2, dynein cytoplasmic 1 light intermediate chain 2; DYNLT3, dynein light chain tctex-type 3; GORASP1, Golgi reassembly stacking protein 1; MAP1A, microtubule associated protein 1A; MAP7D1, MAP7 domain containing 1; MYO1C, myosin IC; STX8, syntaxin 8. One-way ANOVA, *P < 0.05, ***P < 0.001, ****P < 0.0001. (**E**) Live imaging of retention selective hook (RUSH)–synchronized traffic of tumor necrosis factor (TNF) protein in HeLa cells. Scale bars, 10 μm. (**F**) Mean normalized fluorescence intensity (a.u.) after the addition of biotin. Two-stage linear step-up procedure of Benjamini, Krieger, and Yekutieli. See also fig. S7.

To comprehensively assess the function of EXT1 in protein dynamics through the ER, we combined interactome analysis with imaging approaches. First, we captured the EXT1 interactome in the ER microsomes by affinity purification and MS analysis (table S6). Consistent with a role in the ER morphology, spatial analysis of functional enrichment (SAFE) was used to identify three functional modules within EXT1 interactors, two of which were translation initiation and protein targeting to the ER (Fig. 7A). Next, we investigated the potential connections between EXT1 and the secretory pathway by comparing the proteome isolated from control and *EXT1 k.d*. cells after stable isotope labeling by amino acids (SILAC) (Fig. 7B). Differential protein expression analysis indicated the up-regulation of COPII anterograde vesicle-mediated transport components, with concomitant down-regulation of retrograde components following the depletion of EXT1 (Fig. 7, C and D). Thus, we hypothesized that the depletion of EXT1 led to the enhancement of protein recruitment into the luminal ER and subsequent secretion or distribution into cellular membranes.

To further assess the changes in the secretory pathway, we monitored anterograde transport using the retention selective hook (RUSH) system (*33*) that enables the synchronization of cargo trafficking. By tracking cargo transport from the ER to the Golgi using live imaging, we observed a slower dynamic response in *EXT1 k.d*. cells that resulted in an increased residency of the cargo within the secretory pathway (Fig. 7, E and F, and movies S7 and S8). This finding was confirmed using an additional ER export assay based on the vesicular stomatitis virus glycoprotein (VSVG) (fig. S7, A and B) and by examining COPII coat structural components SEC16 and SEC31 (fig. S7, C and D). TEM analysis also indicated a higher number of trans-Golgi secretory vesicles (11.83 ± 7 and 2.4 ± 1.6 secretion vesicles per cell in *EXT1 k.d*. and control cells, respectively) following the depletion of *EXT1* in HeLa cells (fig. S7, E and F). Last, we confirmed enhanced secretion by producing significantly more recombinant proteins (nanoluciferase) in HeLa *EXT1 k.d*. compared to control cells (fig. S7G). The integration of all the above results allowed us to conclude that EXT1 controls secretion by interacting with components of the general translational initiation machinery (Fig. 7A). Thus, EXT1 expression reduction, as modeled by SAFE analysis combining quantitative transcriptional and translational expression (fig. S7H), affects several processes in mammalian cell physiology and metabolism.

## DISCUSSION

Multiple pieces of evidence indicate that EXT1 is broadly implicated in cancer, as suggested by the findings shared in the Cancer Cell Line Encyclopedia, TCGA, and Catalogue of Somatic Mutations in Cancer (*34*–*36*). *EXT1* mutations range from 1% in small cell lung cancer tumors to 27% in colorectal cancers (fig. S1, I and J). At the protein level, we previously reported that EXT1 interacts with Notch1 (*17*). Here, we provide data showing that *EXT1* gene should be considered as an additional regulator molecule involved in T lymphocyte development in mice (Fig. 1, B to E). Furthermore, our results from *EXT1* and *Notch1* double *k.o*. in developing thymocytes demonstrate that cells are able to pass the critical β- and γδ-selection checkpoints in the absence of *Notch1* expression (Fig. 1, D and E). This genetic suppression interaction between *EXT1* and *Notch1* in developing thymocytes reflects a mechanism (exocytosis) potentially controlling T lineage specification, in addition to the well-known transcription and ligand-receptor modulations. Genetic suppression is one of the most powerful tools in yeast (*37*) and *Caenorhabditis elegans* (*38*) genetics. In these organisms, genetic suppression is facilitated by the ability to generate and handle a large number of individual mutations in vivo, allowing global-scale connection of genes involved in the same pathway or biological process. Although systematic examination of *EXT1* genetic interactions was impractical in our mouse models, we demonstrated an overlapping role of *EXT1* and *Notch1* in the developmental stages (DN3 and DN4) of thymocytes and an unexpected healthy phenotype of thymocytes with *EXT1^F/F^ Notch1^F/F^* double k.o. We thus validated a physiological genetic suppression role of *EXT1* in the function of the Notch1 transmembrane receptor. To further demonstrate a potential global role of EXT1 in cancer, we took advantage of the SL (*24*) and the SDL (*25*) principles, whereby, for each pair, individual gene inactivation (SL) or expression variation (SDL) result in viable phenotypes, whereas combined perturbations are lethal. These approaches identified several oncogenes, including *KRAS*, *CREBBP*, *PTEN*, and *BRCA2*, as SDL genetic partners of *EXT1*, highlighting its potential clinical relevance in different cancers.

In vitro the formation of the ER tubular network requires only a small set of membrane curvature and stabilizing proteins (RTNs, REEPs, and large ATL GTPases) (*14*). However, these effectors cannot account for the diversity and adaptability of ER size and morphology observed in individual cell types. It is expected that in vivo, the dynamics of tubular three-way junctions and tubule rearrangements to accommodate luminal flow mobility rely on additional proteins or mechanisms. Despite the discovery of glycoproteins in intracellular compartments 30 years ago (*39*), our knowledge about the glycoproteome is still biased toward secreted and plasma membrane proteins. Glycosylation is well known to regulate the physical properties of different glycolipid and glycoprotein biopolymers at the surface of mammalian cells by controlling plasma membrane and cell coat morphologies (*13*). The central enzyme in the *N*-glycosylation pathway is the OST complex, which catalyzes the transfer of oligosaccharides from dolichol pyrophosphate–linked oligosaccharide to the nascent polypeptides in the protein translocon systems (*40*). The spatial organization of these protein modules is tightly regulated to coordinate temporally coupled synthesis, *N*-glycosylation, and protein translocation (*41*). The atomic structure of yeast OST complex highlighted a potential role of an *N*-glycan at the N539 position of the catalytic subunit STT3 in the stability of the OST complex by sticking together Wbp1 and Swp1–interacting subunits (*31*). Here, we observed that glycosylation of the corresponding residues N548 and N627 of STT3A and STT3B mammalian OST is impaired following the reduction of EXT1. This suggests that EXT1 is involved in the stability of the OST complex in the ER lumen, providing a mechanistic explanation for the lower *N*-glycome observed in *EXT1 k.d*. cells (Fig. 5C). The resulting alternative glycosylation pattern of ER membrane proteins observed here correlated with extensive ER architectural and functional remodeling. Our results suggest that EXT1 is topologically involved in the stability of the OST and the translocon complexes in the ER. Furthermore, the EXT1 interactome revealed here (Fig. 7 and table S6) contains the eucaryotic translation initiation factor 3 (EIF3) subunits E, F, and I; elongation factor 1 (EEF1) α1, β2, and γ subunits; and several 40*S* and 60*S* ribosomal proteins (table S6). A compelling hypothesis following our observations is the possibility that EXT1 coordinates the stability of cotranslationally and posttranslationally *N*-glycosylated proteins in the ER. A potential link between the translasome and efficient protein synthesis and degradation in fission yeast was raised by Sha *et al*. (*42*). However, knowledge of the spatially organization of this supercomplex in mammalian cells is lacking and will require structural studies by cryo–electron microscopy to precisely locate EXT1 relatively to the EIF3-ribosome-EEF1.

The results presented here provide insights into a specific fundamental downstream role of EXT1 in the architecture of the ER. We demonstrated that the reduction of EXT1 affects ER structures, membrane glycome, and lipid compositions, which have broad-ranging metabolic consequences for the cell. We hypothesize that the regulation of ER-membrane macromolecular composition, via alternative glycosylation, reprograms ER trafficking and shape extension, which should enhance cellular productivity, as shown in fig. S7 (E to G). Thus, reprogramming *N*-glycosylation in the ER will have implications in biotechnology including the production of recombinant proteins, therapeutic viral vectors, and vaccines in mammalian cells. As exemplified by the current coronavirus disease 2019 pandemic, insufficient mammalian cell production capacity is a limitation in their industrial use (*43*). EXT1 expression reduction, which affects the structure of the ER, also favors membrane structural fluidity and affects its luminal dynamics. Thus, our findings have demonstrated that glycosylation is an important posttranslational modification controlling the internal plasticity and structure function of the ER. In the future, it will be interesting to identify other glycosyltransferase enzymes that coregulate intracellular organelle morphologies. It is also essential to determine the atomic structure of EXT1 at the ER to clarify the positioning of EXT1 relative to the OST, translocon, and translasome complexes. Together, our findings suggest that the diversity of proteoglycans destined to the cell surface results from the glycosylation equilibrium of intracellular and plasma membrane proteins. At the fundamental level, our findings argue for a general biophysical model of ER membrane extension and functions regulated by resident glycosyltransferase enzymes such as EXT1.

## MATERIALS AND METHODS

### Mice generation

T cell–specific deletion of *EXT1*, *Notch1*, or both genes on a C57BL/6J background was accomplished by intercrossing the EXT1 flox allele (*21*) or Notch1 allele (*23*) and the *lck-cre* transgene (*22*). *EXT1 flox/flox* and Notch1 *flox/flox* mice were a gift from Y. Yamaguchi (Sanford Children’s Health Research Center, Sanford-Burnham Medical Research Institute, La Jolla, CA, USA) and F. Radtke (Ecole Polytechnique Federale de Lausanne, Lausanne, Switzerland), respectively. LCK-CRE [4-8, B6.Cg-Tg (*Lck-cre*) 548Jxm/J] mice were purchased from the Jackson laboratory. T cell–specific depletion of *EXT1* and *Notch1* were verified by polymerase chain reaction (PCR). To detect the deletion of the *EXT1* gene by PCR, the following primers were used on tail tips: BURN-51, 5′-GGAGTGTGGATGAGTTGAAG-3′; BURN-52, 5′-CAACACTTTCAGCTCCAGTC-3′; and BURN-35, 5′-CCAAAACTTGGATACGAGCC-3′. BURN-51 and BURN-52 generate a 460–base pair (bp) fragment from the wild-type allele, and BURN-51 and BURN-35 generate a 509-bp fragment from a CRE-excised allele. To detect *Notch1* deletion by PCR, we used the following primers, generating a 500-bp floxed fragment and 450 bp in wild type: 204 N1 new S, 5′-CTGAGGCCTAGAGCCTTGAA-3′; 205 N1 new AS, 5′-TGTGGGACCCAGAAGTTAGG-3′. We also used the following primers, detecting a deleted band at 400 bp: 15 N1 5′lox, 5′-CTGACTTAGTAG GGGGAAAAC-3′; 17 N1 del3, 5′-AATCAGAGCGGCCCATTGTCG-3′. LCK-CRE mice were used as wild-type controls.

For reverse transcription quantitative PCR (RT-qPCR) on thymocytes isolated from *EXT1^F/F^/lck-cre*, *Notch1^FF^/lck-cre*, *Notch1^F/F^ EXT1^F/F^/lck-cre*, and *lck-cre* control mice, the following primers were used: mEXT1, 5′-GCCCTTTTGTTTTATTTTGG-3′ (forward) and 5′-TCTTGCCTTTGTAGATGCTC-3′ (reverse); mNotch1, 5′-GACACCTCTGGACAACGCCT-3′ (forward) and 5′-CGTGCTCACAAGGGTTGGCAC-3′ (reverse); glyceraldehyde-3-phosphate dehydrogenase (GAPDH), 5′-CCAGTATGACTCCACTCACG-3′ (forward) and 5′-GACTCCACGACATACTCAGC-3′ (reverse). All experiments were performed with mice in the C57BL/6 background. The protocol was approved by the University of Liege ethical committee (authorization #13-1586).

### Mice cell preparation, in vitro T cell activation, and polarization

Single-cell suspensions from the spleen, lymph node, blood, and thymus were obtained by mechanical disruption, straining over a 40-mm nylon mesh, and lysis of erythrocytes. For primary T cell activation, polarization, and cytokine detection of wild-type or *k.o.* mice, isolated CD4^+^ T cells from the spleen and lymph nodes by negative magnetic separation (magnetic-activated cell sorting) using CD4^+^ T cell isolation kit (Miltenyi Biotech) were cultured at a density of 1 × 10^6^ cells/ml on a 96-well plate for 72 hours with biotinylated CD3 and CD28 antibodies. Activation and polarization were performed using the T Cell Activation/Expansion Kit (Miltenyi Biotech), as described in the manufacturer’s instruction manual.

### RNA extraction and RT-qPCR (human cell lines)

Total RNA was extracted using NucleoSpin RNA kit (Macherey-Nagel) according to the manufacturer’s instructions. Real-time qPCR was performed using LightCycler 480 SYBR Green I Master (Roche) and analyzed in triplicate on a LightCycler (Roche). The relative expression levels were calculated for each gene using the ΔΔ*C*t method with GAPDH as an internal control. Primer sequences for qPCR are as follows: EXT1, 5′-GCTCTTGTCTCGCCCTTTTGT-3′ (forward) and 5′-TGGTGCAAGCCATTCCTACC-3′ (reverse); EXT2, 5′-GATTGAAGAAATGCAGAGACAGG-3′ (forward) and 5′-TGGATAGATCCGGTCATTGATA-3′ (reverse); EXTL1, 5′-TGGGCACAGGAAGGTTAGTG-3′ (forward) and 5′-CTTGTGGAAAGACTGCTGCG-3′ (reverse); EXTL2, 5′-ACTCGAGTGACAAGTGAGCC-3′ (forward) and 5′-TGTGGCAACACCTCATTGTG-3′ (reverse); EXTL3, 5′-CAAGAAGTCGTGTGCTGAGG-3′ (forward) and 5′-GCCAACAATCAGGCCATGTG-3′ (reverse); GAPDH, 5′-TTGCCATCAATGACCCCTTCA-3′ (forward) and 5′-CGCCCCACTTGATTTTGGA-3′ (reverse).

### Bromodeoxyuridine proliferation assay

A total of 5 × 10^3^ HeLa shCTRL and shEXT1 cells were seeded in a 96-well plate and cultured overnight. Bromodeoxyuridine (BrdU) was added to the culture medium according to the manufacturer’s instructions (BrdU Cell Proliferation Assay Kit, Cell Signaling), and cells were further incubated for 24, 48, and 72 hours. The absorbance value for each well was measured at 450 nm with a microplate reader TECAN Infinite 200 PRO.

### Proliferation assay using the xCELLigence system

HeLa shCTRL and shEXT1 cells were resuspended in cell culture medium and adjusted to 5000 cells per well. One hundred microliters of each cell suspension was added to the 100 μl of medium-containing wells on E-Plate 96. Cell index was monitored every 3 min for a period of up to 72 hours. The xCELLigence system was used according to instructions supplied by Roche Applied Science and ACEA Biosciences (*44*).

### Subcutaneous xenograft studies

Jurkat cells expressing control luciferase (shLUC or LUC-GFP) and the corresponding shEXT1 or EXT1-LUC-GFP, respectively, were used. Briefly, 2 × 10^6^ viable human cells were mixed with an equal volume BD Matrigel basement membrane matrix and injected into the flanks of 6-week-old sublethally irradiated female NOD-SCID mice. Cell growth and engraftment were monitored every 3 days (Caliper, PerkinElmer). Animals were given an intraperitoneal injection of d-luciferin (150 mg/kg; Promega) and were imaged in groups of up to three mice (for display purposes).

### Transmission electron microscopy

HeLa; HEK293 and Jurkat shCTRL and shEXT1; and HeLa shEXT2, shEXTL1, shEXTL2, and shEXTL3 cells also activated naive CD4^+^ T cells from peripheral lymph organs (spleen and lymph nodes) of EXT1^F/F^; LCK-CRE and LCK-CRE mice that were fixed for 90 min at 4°C with 2.5% glutaraldehyde in Sörensen 0.1 M phosphate buffer (pH 7.4) and postfixed for 30 min with 2% osmium tetroxide. Following dehydration in graded ethanol, samples were embedded in Epon. Ultrathin sections obtained with a Reichert Ultracut S ultramicrotome were contrasted with uranyl acetate and lead citrate. The analysis was performed with a JEOL JEM-1400 transmission electron microscope at 80 kV and in a Tecnai Spirit T12 at 120 kV (Thermo Fisher Scientific, The Netherlands).

### Immunohistochemistry

Immunohistochemical experiments were performed using a standard protocol previously described (*45*). In the present study, the antigen retrieval step was citrate (pH 6.0), and the following primary antibody used was anti-EXT1 (1:50; ab126305, Abcam). The rabbit EnVision kit (Dako) was used for the secondary reaction.

### Flow cytometry, extracellular, and intracellular staining

Single-cell suspensions from the spleen, lymph node, blood, and thymus were prepared as described above. Cells were resuspended in phosphate-buffered saline (PBS) and stained with the following fluorochrome-conjugated monoclonal antibodies purchased from BD Biosciences: anti-mouse CD4–PE (phycoerythrin) CF594 (RM4-5), anti-mouse CD8a-APC (allophycocyanin)-H7 (53-6.7), anti-mouse CD45R/B220-V500 (RA3-6B2), and anti-mouse CD25-BB515 (PC61) for 30 min at 4°C. Cells were washed twice and analyzed by fluorescence-activated cell sorting (FACS). Extracellular stains were performed in PBS supplemented with 0.5% bovine serum albumin and 10% 24G.2 blocking antibody. After polarization, cells were fixed and permeabilized using Foxp3/transcription factor intracellular staining buffer set (eBioscience). The following conjugated monoclonal antibodies were used: anti-mouse IL-17A (interleukin-17A)–efluor450 (eBio17B7), anti-mouse IFN-γ–APC (XMG1.2), and anti-mouse IL-4–APC (11B11). The carboxyfluorescein diacetate succinimidyl ester dye was used to label the dead cells. To quantify HS at the cell surface, 1 × 106 cells are incubated in 100 μl of PBS for 20 min at 37°C. Cells are then washed in PBS and incubated with mouse anti-HS (10E4) for 30 min at 4°C, washed with PBS, and stained with Alexa Fluor 647 secondary anti-mouse antibody for 30 min at 4°C. Cells are analyzed immediately by flow cytometry on a BD LSRFortessa flow cytometer (BD Biosciences).

### Plasmids

HA-SEC13 pRK5 (Addgene #46332), mEmerald-Sec61b-C1 (Addgene #90992), pEGFP-SEC16b (Addgene #66607), pEGFP-SEC23A (Addgene #66609), Str-KDEL-TNF-SBP-mCherry (Addgene #65279), and b4GALT1-pmTirquoise2-N1 (Addgene #36205) constructs were obtained from Addgene. ts045-VSVG-GFP (Addgene #11912) is a gift from F. Heyd (Freie Universität Berlin, Berlin, Germany). EXT1-YFP and Flag-EXT1 were previously described (*17*). Additional cloning vectors used here are as follows: pCSCherryDEST (Addgene #13075), mEmerald-C1 (Addgene #53975), and pSYFP2-C1 (Addgene #22878) or pCS2 EIF ires GFP. The lentiviral constructs used are as follows: shCTRL (anti–enhanced GFP, SHC005, Sigma-Aldrich) or pLV U6 shRNA NT PGK GFP-T2A-Neo, and targeting EXT1 (sh438, TRCN0000039993; sh442, TRCN0000039997; Sigma-Aldrich). The shRNAs targeting EXT2, EXTL1, EXTL2, and EXTL3 were designed using the VectorBuilder online platform and cloned into the lentiviral vector pLV-PURO-U6. The target sequences are listed as follows: EXT2, 5′-AGCGTACTTCCAGTCAATTAAC-3′ or 5′-CCATTGATGATATCATTA-3′; EXTL1, 5′-TGATCGCTTCTACCCATATAG-3′ or 5′-ATACCACTCTGGAGGTTATTC-3′; EXTL2, 5′-CTCTACTTCATCAGGTATCTA-3′ or 5′-GATTCGAGTGCTTCGATTATC-3′; EXTL3, 5′-CCGTACTGAGAAGAACAGTTT-3′ or 5′-TTGCCATTCAAGGCTTATTTA-3′. mCherry-RTN4a, mChery-ATL1, and Lnp1-mCherry lentiviral constructs were a gift from T. Rapoport (Department of Cell Biology, Harvard Medical School, MA, USA). LV-PA-GFP-KDEL is a gift from V. C. Jones (University of Central Lancashire, Preston, UK), and Lenti-ATL3-GFP is a gift from V. Timmerman (University of Antwerp, Antwerp, Belgium). Lentivirus production and instructions on its use were provided by Viral Vectors core facility (Viral Vectors Platform, University of Liege).

### Mammalian cell lines generation and culture

HeLa, HEK293, Jurkat, and Cos7 cells were cultured as previously described (*17*). All stable cell lines were generated by lentiviral transduction. Briefly, HEK293T Lenti-x 1B4 cells (Clontech Lenti-x HEK293T cells) were transfected with calcium phosphate with three plasmids: the vector of interest, pVSV-G (PT3343-5, Clontech), and psPAX2 (Addgene #12260). The supernatants containing the second-generation viral vectors were harvested and concentrated by ultracentrifugation. The cells were transduced with the viral vector of interest with a multiplicity of infection of 50. After 72 hours, the cells were selected for puromycin (InvivoGen) for 3 to 4 days. For fluorescence protein–tagged constructs, positive cells were sorted by flow cytometry.

### DNA-siRNA transfection

DNA was transfected into HeLa and Cos7 with polyethyleneimine (PEI 25K, Polysciences), as previously described (*17*). For siRNA transfection, Cos7 and HeLa cells were transfected at 40 to 50% confluence with 2 nmol of siRNA using a classical calcium phosphate method according to the manufacturer’s instructions (ProFection Mammalian Transfection kit, Promega). The following siRNA duplexes were purchased from Eurogentec (Belgium): siEXT1(1), 5′- GGAUCAUCCCAGGACAGGA-3′; siEXT1(2), 5′-GGAUUCCAGCGUGCACAUU-3′; siCTRL, 5′-GGCUGCUUCUAUGAUUAUGtt-3′.

### Calcium flux detection assay

A total of 2 × 10^5^ Cos7 cells were washed twice and processed for immunofluorescence. Fluo-4, acetoxymethyl (AM) loading solution was added on the cells according to the manufacturer’s instructions (Fluo-4 Calcium Imaging Kit, Thermo Fisher Scientific). Images were acquired using a Leica TCS SP5 confocal microscope and the 63× oil objective; the analysis was performed in ImageJ software.

### Preparation of microsomes from cultured cells

HeLa cells expressing FLAG-EXT1 or HeLa shCTRL and shEXT1 (2 × 10^8^) were harvested and washed with PBS and with a hypotonic extraction buffer [10 mM Hepes (pH 7.8), with 1 mM EGTA and 25 mM potassium chloride] supplemented with a protease inhibitors cocktail. Cells were resuspended in an isotonic extraction buffer [10 mM Hepes (pH 7.8), with 0.25 M sucrose, 1 mM EGTA, and 25 mM potassium chloride] supplemented with a protease inhibitors cocktail and homogenized with 10 strokes using a Dounce homogenizer. The suspension was centrifuged at 1000*g* for 10 min at 4°C. The supernatant was centrifuged at 12,000*g* for 15 min at 4°C. The following supernatant fraction, which is the postmitochondrial fraction (PMF), is the source for microsomes. The PMF was centrifuged for 60 min at 100,000*g* at 4°C. The pellet was resuspended in isotonic extraction buffer supplemented with a protease inhibitor cocktail and stored at −80°C. Isolated membranes were boiled for 5 min in 2× SDS loading buffer. Then, solubilized samples were separated on SDS–polyacrylamide gel electrophoresis (PAGE) and analyzed by Western blotting.

### Western blotting and antibodies

Cells were lysed in immunoprecipitation low-salt buffer [25 mM tris-HCl (pH 7.4), 150 mM NaCl, 1 mM EDTA, 1% NP-40, 5% glycerol, cOmplete Protease Inhibitor (Roche), and Halt Phosphatase Inhibitors (Thermo Fisher Scientific)]. SDS-PAGE and Western blotting were performed using standard protocols. The following primary antibodies were used: mouse anti-Calnexin (1:2000; Abcam), rabbit anti-EXT1 (1:500; Prestige Antibodies, Sigma-Aldrich), mouse anti-NogoA (Santa Cruz Biotechnology), rabbit anti-FLAG (1:4000; Sigma-Aldrich), mouse anti-FLAG (1:4000; Sigma-Aldrich), goat anti-actin (1:2000; Santa Cruz Biotechnology), and rabbit-anti-HSP70 (1:3000; Santa Cruz Biotechnology). Dad1, STT3b, STT3a, Sec61A, Trap-α, TRAP-β, SEC62, and SEC63 rabbit antibodies were a gift from R. Zimmermann (Medical Biochemistry and Molecular Biology, Saarland University, Homburg, Germany). The following conjugated secondary antibodies were used: mouse anti–horseradish peroxidase (HRP) (1:5000; Santa Cruz Biotechnology), rabbit anti-HRP (1:5000; Santa Cruz Biotechnology), and anti-goat (1:5000; Santa Cruz Biotechnology).

### *N*-glycans and *O*-glycans profiling

Microsomes were isolated as described above, and glycans profiling was performed by Creative Proteomics (NY, USA). Briefly, for the preparation of glycans, ~250 μg of lyophilized trypsin- and peptide:N-glycosidase F (PNGase F)–digested protein samples were used in a permethylation reaction. Lyophilized eluted fraction was used for MS analysis. MS data were acquired on a Bruker UltraFlex II MALDI-TOF mass spectrometer instrument. The positive reflective mode was used, and data were recorded between 500 and 6000 mass/charge ratio (*m*/*z*) for *N*-glycans and between 0 and 5000 *m*/*z* for *O*-glycans. For each MS *N*- and *O*-glycan profiles, the aggregation of 20,000 laser shots or more was considered for data extraction. Mass signals of a signal/noise ratio of at least 2 were considered, and only MS signals matching an *N*- and *O*-glycan composition were considered for further analysis and annotated. Subsequent MS post–data acquisition analysis was made using mMass (*46*).

### Glycosyltransferase assay

Glycosyltransferase activity of microsomes from HeLa shCTRL and shEXT1 was determined with the Glycosyltransferase Activity Kit (R&D Systems). A glycosyltransferase reaction was carried out in 50 μl of reaction buffer in a 96-well plate at room temperature for 20 min, according to the manufacturer’s instructions. The absorbance value for each well was measured at 620 nm with a microplate reader Tecan Infinite 200 PRO.

### Metabolomics profiling

For metabolite quantification, HEK293 shCTRL and shEXT1 cells were seeded in triplicate (*n* = 3) in six-well plates with Dulbecco’s modified Eagle’s medium (DMEM) supplemented with 10% fetal bovine serum (FBS). After 24 hours, the medium was removed and replaced with fresh medium containing a stable isotopic tracer ^13^C-glucose. For one well per condition, the medium was replaced with ^12^C-glucose. Upon reaching 70% confluency, the supernatant was stored at −80°C, cells were washed twice with PBS and harvested, and the cell pellet was stored at −80°C until liquid chromatography–MS (LC-MS) identification of metabolites at the University of Leuven metabolomics core facility.

### Lipidomics

Lipidomics analysis was performed in the Lipidomics Core Facility of the University of Leuven, Laboratory of Lipid Metabolism. Briefly, 20 μg of protein or ER microsomes diluted in 700 μl of water was mixed with 800 μl of 1 N of HCl:CH_3_OH (1:8; v/v) and 900 μl of CHCl_3_, in the presence of antioxidant 2,6-di-*tert*-butyl-4-methylphenol (200 μg/ml; Sigma-Aldrich). Three microliters of SPLASH LIPIDOMIX Mass Spec Standard (#330707, Avanti Polar Lipids) was spiked into this mixture, and the samples were vortexed and centrifuged at 4000*g* for 10 min. Lipid species were analyzed by hydrophilic interaction LC electrospray ionization MS/MS on a Nexera X2 ultrahigh-performance LC (UHPLC) system (Shimadzu) coupled with a hybrid triple quadrupole/linear ion trap mass spectrometer (6500+ QTRAP system, AB SCIEX). Chromatographic separation was performed on a XBridge amide column (150 mm by 4.6 mm, 3.5 μm; Waters) maintained at 35°C using mobile phase A [1 mM ammonium acetate in water:acetonitrile, 5:95 (v/v)] and mobile phase B [1 mM ammonium acetate in water:acetonitrile, 50:50 (v/v)] using the gradient (0 to 6 min, 0% B ➔ 6% B; 6 to 10 min, 6% B ➔ 25% B; 10 to 11 min, 25% B ➔ 98% B; 11 to 13 min, 98% B ➔ 100% B; 13 to 19 min, 100% B; 19 to 24 min, 0% B) at a flow rate of 0.7 ml/min, which was increased to 1.5 ml/min from 13 min onward. Lipid quantification was performed by scheduled multiple reaction monitoring (MRM), the transitions being based on the generation of neutral losses or typical fragment ions during collision-induced dissociation in MS/MS. Sphingomyelins (SM), cholesterol esters (CE), ceramides (CER), dihydroceramides (DCER), hexosylceramides (HCER), and lactosylceramides (LCER) were measured in positive ion mode with MRM transitions based on the generation of fragment ions of *m*/*z* of 184.1, 369.4, 264.4, 266.4, 264.4, and 266.4, respectively. Triacylglycerides (TAG), diacylglycerides (DAG), and monoacylglycerides (MAG) were measured in positive ion mode with MRM transitions based on the neutral loss of each of the fatty acyl moieties. Phosphatidylcholine (PC), lysophosphatidylcholine (LPC), phosphatidylethanolamine (PE), lysophosphatidylethanolamine (LPE), phosphatidylglycerol (PG), lysophosphatidylglycerol (LPG), phosphatidylinositol (PI), lysophosphatidylinositol (LPI), phosphatidylserine (PS), and LPS were measured in negative ion mode with MRM transitions based on the neutral loss of each of the fatty acyl moieties.

### Immunofluorescence and confocal, SR microscopy

A total of 3 × 10^4^ Cos7 and 5 × 10^4^ HeLa cells were grown on 18-mm round glass coverslips and transfected with 500 ng of DNA per well. For immunostaining, the cells were washed with PBS (pH 7.4) and fixed with 4% paraformaldehyde in PBS for 15 min at room temperature. Cells were permeabilized with 0.5% Triton X-100 for 10 min and incubated with blocking solution (0.025% Tween 20 and 10% FBS) for 30 min. Primary antibody staining was performed overnight at 4°C in 5% blocking solution: mouse anti–β-catenin (1:1000; RRID:AB_626807), mouse anti-Calnexin (1:500; RRID:AB_2069009), rabbit anti-EXT1 (1:100; RRID:AB_10963838), mouse anti-HS (10E4; RRID:AB_10013601), rabbit anti-GM130 (1:3200; RRID:AB_2797933), mouse anti-PDIA3 (1:1000; RRID:AB_2665750), and mouse anti-SEC31 (1:500; RRID:AB_399716). Goat anti-rabbit, donkey anti-rabbit, or goat anti-mouse secondary antibodies labeled with Alexa Fluor 488 or Texas Red (Thermo Fisher Scientific) and anti-mouse STAR-Red (Abberior) were used at a 1:2000 dilution for 1 hour. Cells were stained with 4′,6-diamidino-2-phenylindole (Thermo Fisher Scientific) when needed for 5 min at room temperature and were mounted with ProLong Antifade Mountants (Thermo Fisher Scientific). Slides were analyzed by confocal microscopy with a Leica TCS SP8 microscope using the 100× oil objective. Images were taken at 2068 by 2068 pixel resolution and deconvoluted with Huygens Professional software. SYFP2-EXT1 was analyzed by STED microscopy with a Leica SP8 STED 592-nm laser. Images were taken at 2068 by 2068 pixel resolution and deconvoluted with Huygens Professional software. SEC31 was analyzed with a Stedycon STED 775-nm laser. mEmerald-EXT1 was analyzed by SR SIM. SIM imaging was performed at the Cell Imaging and Cytometry Core facility (Turku University) using a DeltaVision OMX SR V4 microscope using a 60×/1.42 Olympus Plan Apo N SIM objective and scientific complementary metal–oxide–semiconductor (sCMOS) cameras (Applied Precision), with 2560 by 2160 pixel resolution. The SIM image reconstruction was performed with DeltaVision softWoRx 7.0 software. For live imaging of Cos7 cells expressing mCherry-ATL1 or Lnp1-mCherry, 3 × 10^4^ cells were plated and imaged at 37°C and 5% CO_2_ in a thermostat-controlled chamber on a Zeiss LSM800 AiryScan Elyra S1 SR confocal microscope using the 63× oil objective at 1 frame/100 ms for 5 s. Further analysis was performed in ImageJ software.

### Photoactivatable GFP imaging

A total of 3 × 10^4^ Cos7 cells expressing PA-GFP-KDEL were plated, and live imaging was performed at 37°C and 5% CO_2_ in a thermostat-controlled chamber on a Zeiss LSM800 AiryScan Elyra S1 SR confocal microscope using the 100× oil objective. PA-GFP-KDEL was activated at a perinuclear ER region using the 405-nm laser at 100%, after which the cell was imaged at 1 frame/500 ms for 90 s using the 488-nm laser. Fluorescence intensities were measured using ImageJ software, and data analysis and curve fitting were performed in GraphPad Prism 8 (GraphPad Software). To avoid intercell variability, the activation site was at the perinuclear area of cells with the same ER density. The integrated fluorescence intensity of each region of interest (ROI) at fixed distances (8, 12, and 16 μm) from the activation region was measured in ImageJ. Normalization of raw values was performed by defining the initial fluorescence to 0 and the maximum fluorescence to 1 for each ROI. Image analysis was performed in ImageJ.

### Affinity purification and MS

Solubilization buffer (2×) [3.5% digitonin, 100 mM Hepes (pH 7.5), 800 mM KOAc, 20 mM MgOAc_2_, and 2 mM dithiothreitol] was mixed in a ratio 1:1 with the microsomal fraction and incubated 10 min on ice. Equilibrated agarose beads M2-FLAG (Sigma-Aldrich) were added in the microsomal fraction (15 μl of beads per half of a 10-cm cell culture dish), and rotation was performed overnight at 4°C. Beads were washed three times for 15 min with glycine of 50 mM (pH 3.0) for protein elution. The supernatant was supplemented with tris-HCl (pH 8.0). Eluted proteins were then subjected to trypsin digestion and identified by MS.

For glycopeptide identification, the resulting MS/MS data were processed using Byonic 3.5 (Protein Metrics) search engine within the Proteome Discoverer 2.3 against a human database obtained from UniProt; the glycan database was set to “*N*-glycan 182 human no multiple fucose or *O*-glycan 70 human.” Trypsin was specified as cleavage enzyme, allowing up to two missed cleavages, five modifications per peptide, and up to seven charges. Mass error was set to 10 parts per million (ppm) for precursor ions and 20 ppm for fragment ions. Oxidation on Met, carbamidomethyl (+57.021 Da) was considered as variable modifications on Cys. Glycopeptides with a Byoinic score of ≥300 and with a Log Prob of ≥4.0 were retained, and their identification was manually validated.

### SILAC labeling

HeLa cells (shCTRL and shEXT1) were cultured for at least five cell doublings in either isotopically light or heavy SILAC DMEM obtained from Thermo Fisher Scientific (catalog number A33969) containing 10% FBS and streptomycin (50 μg/ml) and penicillin (50 U/ml) (Lonza). For the heavy SILAC medium, 50 mg of ^13^C_6_ l-lysine–2HCl (heavy) and 50 mg of l-arginine–HCl were added. In light SILAC medium, 50 mg of l-lysine–2HCl (light) and 50 mg of l-arginine–HCl were added. A total of 2 × 10^5^ cells were adapted to grow in DMEM. The cell pellet was suspended in 150 μl of modified radioimmunoprecipitation assay (RIPA) buffer and sonicated, followed by incubation at 60°C for 15 min. Samples were clarified by centrifugation; each replicate was pooled and quantified by Qubit (Invitrogen): 20 μg of the sample was separated on a 4 to 12% bis-tris Novex minigel (Invitrogen) using the Mops buffer system. The gel was stained with Coomassie, and gel bands were excised at 50 and 100 kDa. Gel pieces were processed using a robot (ProGest, DigiLab). They were washed with 25 mM ammonium bicarbonate followed by acetonitrile, reduced with 10 mM dithiothreitol at 60°C followed by alkylation with 50 mM iodoacetamide at room temperature, and digested with trypsin at 37°C for 4 hours. Last, they were quenched with formic acid, and the supernatant was analyzed directly without further processing. For the SILAC analysis performed by MS Bioworks LLC (MI, USA), the samples were pooled 1:1, and 20 μg was separated on a 4 to 12% bis-tris Novex minigel (Invitrogen) using the Mops buffer system. The gel was stained with Coomassie, and the lanes were excised into 40 equal segments using a grid. For MS, the gel digests were analyzed by nano–LC-MS/MS with a Waters NanoAcquity HPLC system interfaced to a Thermo Fisher Scientific Q Exactive. Peptides were loaded on a trapping column and eluted over a 75-μm analytical column at 350 nl/min. Both columns were packed with Luna C18 resin (Phenomenex). Data were processed through the MaxQuant software 1.5.3.0 (www.maxquant.org), which served several functions such as the recalibration of MS data, the filtering of database search results at the 1% protein and peptide false discovery rate, the calculation of SILAC heavy/light ratios, and data normalization.

### Rush assay

An adaptation of the published assay (*33*) was used. HeLa cells were transfected with Str-KDEL-TNF-SBP-mCherry construct as described above, and 24 hours after transfection, mCherry-positive cells were sorted. A total of 5 × 10^4^ cells were cultured on 35-mm imaging dish. The day after, cells were transferred at 37°C in a thermostat-controlled chamber. At time point zero, the medium was removed and replaced with medium containing d-biotin (Sigma-Aldrich) at 40 μM concentration. The time-lapse acquisition was made using a Zeiss LSM800 AiryScan Elyra S1 SR confocal microscope. Images were acquired using a 63× oil objective. For each time point, the integrated intensity of an ROI was measured. The integrated intensity of an identical-size ROI corresponding to background was measured and subtracted from the values of the integrated intensity for each time point. The values were then normalized to the maximum value. These quantifications were performed using the Zeiss Black software.

### Export assay

A total of 3 × 10^4^ Cos7 cells were cultured on 35-mm imaging dish and transfected with the ts045-VSVG-GFP reporter construct and immediately incubated at 40°C overnight to retain the reporter protein in the ER. After the addition of cycloheximide, cells were transferred in a thermostat-controlled chamber at 40°C. The temperature was shifted to 32°C, and cells were processed for immunofluorescence at *t* = 0, *t* = 45, and *t* = 90 min and stained with mouse anti–β-catenin antibody as described above. The acquisition was made using a Zeiss LSM800 AiryScan Elyra S1 SR confocal microscope. Images were acquired using a 40× oil objective.

### Image analysis

For colocalization analysis, the average Pearson’s correlation coefficient test was performed with the plugin Colocalization Threshold in ImageJ software. To track the displacement of main junctions during successive frames, the dynamic features of the cell were retrieved from the time lapses of Cos7 cells expressing mCherry-ATL1 or Lnp1-mCherry with the following image processing procedure. Images were preprocessed to uniformize the intensities. Then, each image was binarized and skeletonized using MATLAB 2016a. The skeleton was labeled using the AnalyzeSkeleton plugin from ImageJ. From this process, each pixel of the skeleton was classified according to its neighborhood, leading to three-pixel classes: end point, junctions, and tubules. To reflect the structure of the ER, the ratio of the junctions over the tubules was computed for mCherry-ATL1 and Lnp1-mCherry proteins. The dynamics of the ER was assessed by the main junction displacement during a time lapse. To achieve the tracking of the displacement, the junctions larger than three pixels were kept segmented. Then, the segmented objects were multiplied by the initial image intensity to consider the initial light intensity. Last, a Gaussian blur was applied to these objects. The tracking of the bright spot was achieved using a single-particle tracking algorithm, the “simple LAP tracker” available in ImageJ plugin TrackMate. The parameters were set following the recommendations for Brownian motion–like movements, i.e., a max linking distance of 7 pixels, a max closing distance of 10 pixels, and a max frame gap of 3 pixels. From the results of TrackMate, only the tracks longer than 10 frames were kept to reduce the noise. Last, using all velocity vectors measured, a cumulative velocity distribution was computed. Furthermore, a diffusion coefficient based on instantaneous velocity was computed using the MATLAB as described previously (*32*).

In the AnalyzER software (*47*), original images were imported, and the ROIs were segmented using Otsu’s method. Cisternae are identified using an image opening function and active contour refinement. The tubular network is enhanced using phase congruency, and the resulting enhanced network is skeletonized to produce a single-pixel-wide skeleton running along each tubule. Regions fully enclosed by the skeletonized tubular network and the cisternae are defined as polygonal regions, and features such as area, circularity, and elongations are extracted.

### SAFE analysis

We used the SAFE software (v1.5) to determine and visualize significant functional modules (i) in the network of EXT1 partners and their first-order neighbors excerpted from the STRING database with confidence over 0.95 and (ii) in the network of genes whose expressions are significantly regulated by EXT1 k.d. obtained from the STRING database with confidence over 0.9. The network layouts were generated with Cytoscape (v3.4.0) using the edge-weighted spring-embedded layout. Gene ontology (GO) terms for each gene were extracted from FuncAssociate (v3; GO updated on February 2018). The SAFE analysis was run with the default option.

### RNA sequencing

RNA sequencing analysis was previously described and is deposited as GSE138030.

### Model generation and FBA

Model generation and in silico FBA were performed using the COBRA toolbox V3.0 in the MATLAB 2018a environment with an interface to IBM Cplex and GNU Linear Programming Kit (GLPK) solvers provided in the COBRA toolbox. Linear programing problems were solved on a macOS Sierra version 10.12.6. To generate the control and EXT1 k.d.–specific models, the gene expression mRNA data for samples of control EXT1 k.d. cells (RNA sequencing) were integrated with the COBRA human model, *RECON2*. The integration step uses the GIMME (gene inactivity moderated by metabolism and expression) algorithm, available in the COBRA toolbox. Because GIMME requires binary entries for the indication of the presence or absence of genes, we used a gene expression threshold value equals to the first quartile RPKM (reads per kilobase of transcript per million) for genes in control and EXT1 k.d. cells. GIMME only integrates reactions associated with active genes, leaving those associated with the lowly expressed genes inactive. Therefore, genes with expression values below the threshold were given the value of 0 (inactive), and those with expression values higher than the threshold were given a value of 1 (active). FBA calculates the flow of metabolites through a metabolic network, thereby predicting the flux of each reaction contributing to an optimized biological objective function such as growth rate. Simulating growth rate requires the inclusion of a reaction that represents the production of biomass, which corresponds to the rate at which metabolic precursors are converted into biomass components, such as lipids, nucleic acids, and proteins. For both models generated after the integration step, we used the biomass objective function as defined in the *RECON2* model to obtain the FBA solution using the COBRA Toolbox command, *optimizeCbModel.* After identification of the objective function in the model, the entries to the command *optimizeCbModel* are as follows: the model and the required optimization of the objective function (maximum production). The command output is the FBA solution, which includes the value of the maximum production rate of the biomass and a column vector for the conversion rate value (reaction fluxes) of each metabolite accounted for in the model.

### Statistical analysis

Graph values are represented as means ± SD of the mean calculated on at least three independent experiments/samples. The analyses were performed in Prism 8 (GraphPad Software). The statistical significance between means was determined using one-way analysis of variance (ANOVA), followed by two-tailed, unpaired Student’s *t* test. *P* value thresholds are depicted as follows: *P < 0.05, **P < 0.01, ***P < 0.001, and ****P < 0.0001; n.s. indicates not significant. Significance for PA-GFP-KDEL was performed using two-way ANOVA, followed by Sidak’s multiple comparisons test. Significance for Rush assay was performed using the two-stage linear step-up procedure of Benjamini, Krieger, and Yekutieli, with *Q* = 1%. Each time point was analyzed individually, without assuming a consistent SD.

## SUPPLEMENTARY MATERIALS

Supplementary material for this article is available at http://advances.sciencemag.org/cgi/content/full/7/19/eabe8349/DC1

## Appendix

View/request a protocol for this paper from *Bio-protocol*.
